# Audiovisual Speech Recognition in Adult Cochlear Implant Candidates: Relations with Cognitive-Linguistic Abilities

**DOI:** 10.1097/ONO.0000000000000082

**Published:** 2026-01-07

**Authors:** Gabriella Cote, Gizem Babaoglu, Morgan A. Zupkus, Hugh M. Birky, Michayla M. Saraino, Jonathan D. Neukam, Terrin N. Tamati, Aaron C. Moberly

**Affiliations:** 1 Department of Otolaryngology–Head and Neck Surgery, Vanderbilt University Medical Center, Nashville, TN.

**Keywords:** Audiovisual speech recognition, Cochlear implants, Sensorineural hearing loss, Short-term memory, Speech recognition

## Abstract

**Objective:**

To examine audiovisual (AV) sentence recognition performance in adults with moderate-to-profound sensorineural hearing loss who are cochlear implant (CI) candidates, and to identify the demographic, audiologic, and cognitive-linguistic abilities that explain individual differences in AV performance.

**Study Design:**

Cross-sectional observational study.

**Setting:**

Tertiary referral center.

**Patients:**

Twenty-four postlingually deafened adults (mean age = 70.3 years, SD = 11.2) meeting clinical CI candidacy criteria in both ears, with no known history of cognitive or neurological disorders.

**Intervention(s):**

Participants completed the Multimodal Lexical Sentence Test (MLST) for Adults for AV sentence recognition and a battery of cognitive-linguistic tasks measuring short-term memory, working memory, inhibition-concentration, nonverbal reasoning, and speed of lexical/phonological access.

**Main Outcome Measure(s):**

AV sentence recognition accuracy on the MLST.

**Results:**

MLST performance varied broadly across participants (range = 11%–98%, mean = 67.6, SD = 29.7) and was strongly correlated with auditory-only sentence recognition abilities (*rho* = 0.85, *P* < 0.001). Otherwise, AV sentence recognition was not significantly correlated with demographic or additional audiological variables. Short-term memory capacity, measured using visual Forward Digit Span, significantly explained MLST performance (partial *rho* = 0.44, *P* = 0.02), after controlling for auditory-only sentence recognition in quiet. Other cognitive-linguistic measures were not significantly associated with MLST scores.

**Conclusions:**

AV sentence recognition in adult CI candidates is partially explained by short-term memory capacity. Ecologically valid AV assessments may help identify cognitively mediated barriers to real-world communication that are not captured using conventional auditory-only speech recognition tests.

Cochlear implants (CIs) are a transformative intervention for adults with moderate-to-profound sensorineural hearing loss (SNHL), improving speech recognition for many. However, CI users show substantial outcome variability that is not fully explained by demographic, audiological, and surgical factors (1–3). This variability appears even more pronounced in real-world settings (4). CI users frequently report difficulty in noisy, multi-talker environments—especially when visual cues are limited (5). Conventional clinical auditory-only sentence recognition measures (eg, AzBio) offer limited insight into real-world experiences, as they assess speech perception under idealized, unisensory conditions in the sound booth that fail to capture the multisensory and cognitive demands of everyday communication (6,7).

Much of real-world everyday speech perception is inherently audiovisual (AV) in nature. During in-person interactions, listeners integrate visual speechreading cues—such as lip movements and facial expressions—with auditory cues to facilitate understanding (8). Visual cues are especially important in challenging listening environments that include background noise, degraded auditory signals, or video-mediated communication (eg, Zoom). Grant and colleagues (9) demonstrated that adding visual speech cues significantly improved sentence recognition scores for individuals with SNHL when auditory signals were degraded. Similarly, Kushalnagar and Volger (10) emphasized that deaf and hard-of-hearing individuals rely heavily on visual information during videoconferencing. These findings underscore the role of visual input in maintaining speech understanding in real-world environments for individuals with hearing loss.

Research suggests that many individuals with SNHL—including CI users and CI candidates—adapt to auditory deprivation by increasing their reliance on visual speech cues, through enhanced AV integration (11). AV integration refers to the brain’s capacity to combine auditory and visual inputs to support speech recognition, particularly under degraded or noisy listening conditions (12). This process involves not only the extraction of acoustic and visual cues from speech signals but also their integration and interpretation using higher-level phonological, syntactic, and semantic knowledge. Rouger and colleagues demonstrated that adult CI users performed better than normal-hearing (NH) peers on visual-only speech tasks, consistent with a shift toward visual compensatory strategies (11).

Still, the benefits derived from AV input by postlingually deafened adults vary widely. Moberly and colleagues (13) found that AV speech recognition performance in adults with CIs and in NH peers listening to noise-vocoded speech (ie, stimuli processed in a fashion similar to how a CI processes speech) was highly variable. In that study, AV sentence recognition performance was related to inhibition-concentration skills and vocabulary knowledge in NH adults but not CI users. This discrepancy may reflect the different demands placed on cognitive control when interpreting temporarily degraded speech by NH listeners versus CI users who were accustomed to hearing degraded input. These findings suggest that successful AV speech perception under degraded listening conditions relies not only on access to visual cues but also on particular cognitive-linguistic abilities. In individuals with mild-to-moderate SNHL, Frei and colleagues (14) found that AV sentence recognition—measured using speech in speech-shaped noise at a 0 dB SNR—was significantly related to working memory. Thus, neurocognitive abilities may support flexible AV cue integration under degraded listening conditions.

Further supporting the importance of AV processing abilities in adults with moderate-to-profound SNHL, Moberly et al (15) found that CI candidates with stronger AV integration before implantation achieved better auditory-only speech recognition performance 6 months after implantation (15). This finding highlights the potential value of assessing AV speech processing even before implantation and raises important questions about which cognitive and linguistic skills support success on ecologically valid speech perception tasks both before and after cochlear implantation.

The purpose of the current study was to examine the AV speech recognition performance of adults with moderate-to-profound SNHL who are CI candidates, and to determine cognitive and linguistic abilities that can explain variability in AV performance. We used the Multimodal Lexical Sentence Test (MLST) to evaluate AV speech processing (16). The MLST incorporates visual speech cues, talker variability, and a range of lexical difficulty to simulate aspects of real-world communication, making it a more ecologically valid tool for assessing individual differences in AV speech perception (17). The hypotheses were that CI candidates would demonstrate broad variability in MLST performance and that cognitive-linguistic measures of short-term memory, working memory, inhibition-concentration, nonverbal reasoning, and speed of lexical and phonological processing would explain MLST performance above and beyond auditory-only speech recognition abilities. We also explored whether individual differences in AV sentence recognition were related to typical demographic variables, such as age, socioeconomic status (SES), and duration of hearing loss. While this study focuses on adult CI candidates, we adopt a broader lens: these individuals represent a larger population of adults with moderate-to-profound SNHL—many of whom rely heavily on hearing aids and compensatory strategies to try to remain communicatively active. Investigating performance of adults with moderate-to-profound SNHL on ecologically valid speech perception tasks like the MLST can offer deeper insight into the cognitive and perceptual mechanisms that underlie communication in everyday life.

## MATERIALS AND METHODS

### Participants

A total of 24 adults between the ages of 43 and 83 years (mean = 70.3, SD = 11.2) were enrolled and underwent research testing before cochlear implantation. Participants were recruited from a large CI center after being determined clinically to meet traditional candidacy criteria for cochlear implantation. Inclusion criteria included bilateral moderate-to-profound SNHL and ≤60% correct on AzBio sentence recognition testing in quiet or at +5 dB SNR in multi-talker babble under best-aided conditions in each ear separately during the clinical CI evaluation appointment (18). All were native English speakers. Exclusion criteria included prelingual deafness, inner ear malformation or ossification on preoperative imaging, history of stroke or neurological disorder, or previously diagnosed cognitive impairment.

Screening assessments included a visually administered Mini-Mental State Examination (MMSE), with a required score of≥24 indicating normal cognitive function. Visual acuity was assessed with a near-vision screening examination, with corrected near vision of at least 20/40 required. Participants also completed the Word Reading subtest of the Wide Range Achievement Test (WRAT), Fourth Edition, to assess general language proficiency (19). Only participants with WRAT standard scores indicating adequate reading ability (≥75) were included. SES was assessed using a validated metric based on occupational and educational levels, resulting in scores from 1 to 64 (lowest to highest, respectively) (20). Additional demographic and audiological variables were collected, including better-ear unaided pure-tone average (BE-PTA, across 500, 1000, and 2000 Hz), hearing loss duration (current age minus self-reported age at onset of hearing loss), and preoperative sentence recognition using AzBio sentences in quiet (AzBioQ) (18), presented at 65 dB sound pressure level (SPL) in everyday listening configuration, meaning with their own hearing aid(s) if typically used and without hearing aids if not typically worn. More specifically, 14 participants were tested with their own bilateral hearing aids, 4 were tested with a unilateral hearing aid, and 6 were tested without hearing aids. Importantly, AzBioQ sentence recognition performance was collected in the same listening configuration and presentation level as MLST performance.

### General Approach and Measures

Participants were tested in one session lasting approximately 4 hours with breaks, as part of a larger study on adult CI outcomes. All tasks were performed in a soundproof booth or sound-treated testing room. Participants completed the AV sentence recognition task and a battery of nonauditory cognitive and linguistic measures. Participants were tested in their everyday listening configuration, as defined above. For tasks requiring a spoken participant response, responses were audio-recorded to allow later offline scoring. Two experimenters independently scored 25% of responses to assess reliability. For computerized tasks, participants entered responses directly into the computer, which generated scores. Participants provided informed written consent and received $15 per hour for their time. Local Institutional Review Board approval was obtained.

### Audiovisual Speech Recognition

AV sentence recognition was assessed using the MLST for adults, a validated prerecorded test of AV speech perception (16,17). Stimuli were sentences produced by a single Caucasian female talker, selected based on findings from Lewis and colleagues (17), which demonstrated that this talker yielded the highest intelligibility scores in vocoded conditions (6- and 8-channel noise vocoding) when tested with NH listeners.

MLST sentence materials were lexically balanced and varied in both word frequency and phonological neighborhood density. Only AV stimuli were used in the current study. Participants were seated approximately 1 meter from a loudspeaker at 0° azimuth, presented at 65 dB SPL, with visual stimuli displayed on a 39-inch monitor directly below the speaker. During each trial, participants viewed a dynamic video of the talker’s face while listening to the auditory component. A total of 30 sentences were presented in 1 block. Participants were instructed to repeat each sentence aloud verbatim. Responses were recorded and later scored offline for the percentage of total words correctly repeated.

### Cognitive-Linguistic Measures

Participants completed 4 visual cognitive-linguistic tests, with task order counterbalanced to mitigate fatigue and order effects. All tasks were administered in a quiet, well-lit room. Measures were selected to reflect cognitive-linguistic capacities previously shown to influence speech recognition under degraded or multisensory conditions (13,14).

1. Nonverbal reasoning was assessed using a computerized version of Raven’s Progressive Matrices, which uses nonverbal, pattern-based puzzles to measure abstract reasoning and problem-solving abilities (21). Participants completed as many items as possible in a 10-minute abbreviated version of the task. Each item consisted of a geometric matrix with a missing piece; participants selected the option that completed the pattern. Raw score (total items correct) was used in the analysis.
2. Short-term and working memory were assessed using the forward digit span (FDS) and backward digit span (BDS), respectively. A visual digit span task was adapted from the Wechsler Intelligence Scale for Children – Fourth Edition (22). In this version, digit sequences were presented visually, 1 digit at a time, and participants reproduced the sequences in either forward (FDS) or backward (BDS) order. In both parts, the sequence length increased adaptively based on correct responses. The total number of digits correctly recalled in appropriate order before making 2 consecutive errors served as the total correct score for each condition.
3. Inhibition-concentration was assessed using a computerized visual Stroop task (Millisecond Software) (23). Participants were shown color words (eg, “green”) in colored fonts on a screen that were either congruent (eg, the word “blue” in blue ink), incongruent (eg, the word “red” in green ink), or neutral (a colored rectangle with no text). They were instructed to identify the color of the font by pressing a corresponding color-coded key. Each condition included 28 trials in random order. Response times were recorded for correct responses. The mean response time for incongruent trials was treated as the score of inhibition, while the mean response time for control trials was treated as the score of concentration. Longer response times reflect poorer performance.
4. Lexical and phonological access speed were measured using the Test of Word Reading Efficiency, Version 2 (24). Participants were asked to read aloud as many real words (108) and pseudowords (66) as possible within 45 seconds. Number of correct words for each condition (words, representing lexical access speed, or nonwords, representing phonological access speed) was used in analyses.

### Data Analysis

Analyses were performed using SPSS version 29.0.2.0 (IBM Corporation, Chicago, IL). To account for the non-normal distribution of MLST scores, Spearman’s rank order correlations were performed between MLST performance and demographic-audiologic variables, as well as partial rank order correlations between MLST performance and each cognitive-linguistic measure, while controlling for AzBioQ performance. Controlling for AzBioQ was done with the expectation that individuals with better everyday aided AzBioQ performance would score higher on the MLST based on better access to auditory-only speech information. This approach allowed us to examine how=c abilities relate to AV sentence recognition independently of hearing and auditory-only sentence recognition abilities. One-tailed analyses were used based on the hypothesis that better cognitive-linguistic performance would correlate with better MLST performance. Effect sizes for Spearman partial correlation coefficients were defined by Cohen: small effects (Spearman coefficient 0.10–0.29), medium effects (Spearman coefficient 0.30–0.49), and large effects (Spearman coefficient >0.50) (25). For all analyses, an alpha of 0.05 was set. Partial correlation coefficients are reported, with a Holm–Bonferroni correction applied to correct for multiple comparisons.

## RESULTS

### Participant Characteristics

Descriptive statistics for demographic and audiologic variables are provided in Table 1. Participants’ mean age was 70.3 years (SD = 11.2), and the mean BE-PTA was 74.8 dB HL (SD = 16.7). Mean preoperative everyday AzBioQ sentence recognition score was 52.8% (SD = 34.0). Performance for all participants on the MMSE was normal, except for 1 participant who did not complete the MMSE due to time constraints (but achieved approximately the mean cognitive-linguistic scores, so was included in analyses). For the WRAT, mean performance was at least 75, except for 4 participants whose audio response recordings were lost due to a technical error (but achieved approximately mean cognitive-linguistic scores, so were included in analyses). One participant did not report occupation, so SES could not be computed.

**TABLE 1.** Participant characteristics

	N	Mean	SD
Age (years)	24	70.3	11.2
MMSE (raw score)	23	28.2	1.9
WRAT Scaled Score	20	95.2	9.4
SES	23	33.1	16.4
Better-ear PTA (dB HL)	24	74.8	16.7
Duration of hearing loss (years)	24	32.9	22.2
AzBioQ (% correct)	24	52.8	34.0

AzBioQ indicates AzBio sentence recognition score in quiet; dB HL, decibels hearing level; MMSE, Mini-Mental State Examination; PTA, pure-tone average; SES, Socioeconomic status; WRAT, Wide Range Achievement Test word reading score.

### Multimodal Lexical Sentence Test Audiovisual Performance and Demographic-Audiologic Variables

MLST performance ranged from 11% to 98% (mean = 67.6, SD = 29.7), indicating broad individual differences in AV sentence recognition. As shown in Table 2, as expected, MLST performance was strongly correlated with AzBioQ sentence recognition (*rho* = 0.85, *P* < 0.001). Otherwise, none of the other Spearman correlations between MLST and demographic or audiologic variables, including age, duration of hearing loss, SES, or BE-PTA, were significant. BE-PTA showed an effect that was close to significant (*P* = 0.09), and the effect size for its correlation with MLST performance was only small in magnitude (*rho* = −0.28), with worse BE-PTA weakly correlating with poorer MLST scores. Altogether, these findings provided support for controlling for AzBioQ scores in our next set of analyses.

**TABLE 2.** Spearman correlations of Multimodal Lexical Sentence Test audiovisual sentence recognition performance and demographic-audiologic variables

	Spearman *rho* correlation coefficient	*P* value
Age (years)	−0.13	0.28
Duration of hearing loss (years)	−0.01	0.48
SES	0.18	0.21
Better-ear PTA (dBHL)	−0.28	0.09
AzBioQ (% correct)	0.85	<.001

AzBioQ indicates AzBio sentence recognition score in quiet dB HL, decibels hearing level; PTA, pure-tone average; SES, Socioeconomic status.

### Multimodal Lexical Sentence Test Audiovisual Performance and Cognitive-Linguistic Variables, Controlling for AzBioQ Performance

Partial Spearman correlations were performed between MLST scores and cognitive-linguistic variables, controlling for AzBioQ performance. Results are reported in Table 3. FDS was significantly associated with MLST performance (partial *rho* = 0.44, *P* = 0.02) while controlling for AzBioQ performance, with a medium effect size, and this remained significant after Holm–Bonferroni correction. All other cognitive-linguistic variables were not significantly associated with MLST performance while controlling for AzBioQ performance.

**TABLE 3.** Spearman partial correlations of Multimodal Lexical Sentence Test audiovisual sentence recognition with cognitive-linguistic variables, controlling for AzBio sentence recognition performance in quiet

	Spearman *rho* partial correlation	*P* value
Ravens Matrices (total words correct)	0.19	0.19
FDS (total correct)	0.44	0.02^a^
BDS (total correct)	0.22	0.17
Stroop control response time (msec)	−0.29	0.10
Stroop incongruent response time (msec)	−0.07	0.37
TOWRE words (percent correct)	0.22	0.18
TOWRE nonwords (percent correct)	0.18	0.23

a Significant after Holm–Bonferroni correction.

BDS indicates backward digit span; FDS, forward digit span; msec, milliseconds; TOWRE, Test of Word Reading Efficiency.

**FIG. 1.**
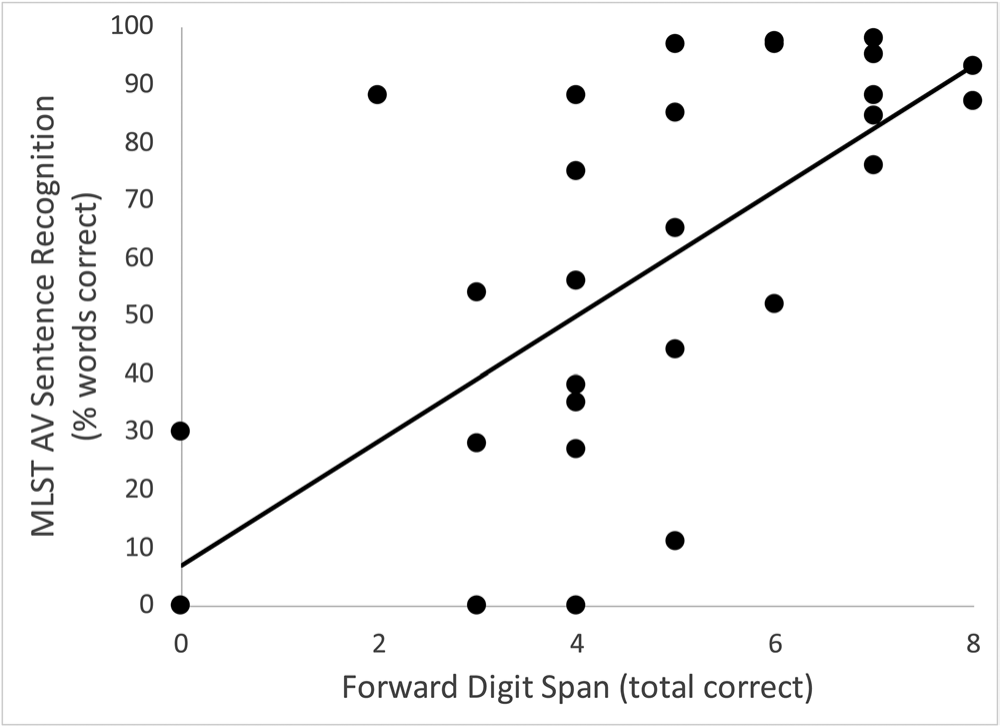
Scatterplot and regression line of audiovisual (AV) sentence recognition performance on the Multimodal Lexical Sentence Test for Adults (MLST) versus visual Forward Digit Span.

To visualize individual differences in MLST and FDS performance, a scatterplot displaying these scores by participant is shown in Figure 1. This figure demonstrates the findings of our analyses above, with a relationship between better FDS performance and better MLST performance, with this relationship remaining significant when controlling for AzBioQ auditory-only sentence recognition performance.

## DISCUSSION

This study aimed to examine AV speech recognition performance in adults with moderate-to-profound SNHL who were candidates for cochlear implantation, and to identify the cognitive-linguistic abilities that explain variability in performance. As hypothesized, participants demonstrated broad variability in AV sentence recognition as measured using the MLST. As expected, MLST performance was strongly correlated with the everyday listening configuration of AzBioQ auditory-only sentence recognition, although we are not aware of other studies that have specifically assessed this relationship. Beyond that relationship, AV sentence recognition variability was not explained by demographic variables such as age, SES, or duration of hearing loss, and was only weakly (and not significantly) correlated with BE-PTA.

The broad range of MLST performance observed aligns with prior work documenting significant individual differences in AV speech recognition among CI candidates (13,15). Such variability may be even greater in naturalistic environments, where listeners must process multimodal input under conditions of noise, reverberation, and talker variability (6,8,14). The MLST incorporates dynamic AV cues and a range of lexical difficulty (16,17), making it a more ecologically valid measure of speech recognition than traditional auditory-only tests.

Our findings are consistent with prior research highlighting the role of cognitive resources in supporting AV speech comprehension, above and beyond auditory-only speech recognition abilities. Frei and colleagues (14) examined AV speech comprehension in older adults with mild-to-moderate SNHL using a continuous speech-in-noise task that required participants to match sentences to written target probes. This task emphasized comprehension rather than simple word identification, placing demands on working memory. Crucially, adding visual cues did not significantly improve average performance across participants. This absence of benefit was attributed in part to the complexity of the task, which required listeners to store, compare, and comprehend longer stretches of speech. The authors argued that AV input can increase cognitive and perceptual load for some listeners. However, individual differences in working memory (WM) capacity still predicted better performance in AV conditions as compared with auditory-only co=gesting that listeners with greater cognitive resources were better able to benefit from visual cues.

Our findings identified short-term memory (STM), more than WM capacity, as the strongest cognitive predictor of AV sentence recognition when accounting for auditory-only sentence recognition abilities using AzBioQ performance. Specifically, performance on the FDS task—a measure of STM—emerged as a significant predictor of MLST accuracy with a medium effect size (*rho* = 0.44), suggesting that the ability to temporarily store and sequence linguistic input is critical for successful recognition when auditory signals are highly degraded, as is typical for CI candidates. In contrast, the other cognitive-linguistic skills assessed, including working memory, inhibition-concentration, nonverbal reasoning, and speed of lexical and phonological processing, did not show significant relationships with MLST performance. Still, the trend is consistent with prior research, which has shown that either STM or WM capacity may play a role in AV speech perception (14). Our findings support the broader view that AV speech perception is cognitively mediated and shaped by both the severity of auditory degradation and the demands of the listening task (26). The authors of that prior study characterized AV integration as a context-sensitive process supported by flexible, distributed networks—including attentional, memory, and language systems—that are differentially engaged depending on task complexity (13,14,25).

The MLST task provides a more ecologically valid assessment of speech recognition than traditional auditory-only tests. Unlike sentence recognition tests such as the clinically used AzBio test, which evaluates speech processing under relatively idealized, auditory-only conditions, the MLST presents dynamic AV speech with natural sentence structure and lexical variability (6). This task better approximates real-world conversation, in which listeners integrate multiple sources of information across auditory and visual modalities (8,9,12). Individuals with hearing loss frequently report that visual cues are essential for understanding speech in everyday settings—especially when noise or reverberation additionally interferes with auditory input (10,14).

The sentence-based format of the MLST likely enabled listeners to draw on top-down syntactic and semantic cues to facilitate lexical access. Walden and colleagues (27) noted that sentence context provides constraints that reduce ambiguity and support word recognition—particularly under degraded listening conditions. This may help explain why FDS contributed as a factor associated with MLST performance: sentence recognition in multimodal tasks may rely more on the temporary retention, sequencing, and repetition of linguistic input above and beyond the requirements of auditory-only testing.

From a theoretical perspective, findings are broadly consistent with the Ease of Language Understanding model, which suggests that when sensory input fails to match stored phonological representations—such as in cases of hearing loss or listening in noise—comprehension shifts from automatic to effortful, relying on top-down cognitive support (28). In our sample, degraded auditory input may have necessitated greater reliance on cognitive scaffolding to integrate visual cues and reconstruct sentence meaning. Our finding that STM emerged as a predictor of AV sentence recognition may reflect the demands of temporarily storing and sequencing linguistic input. This interpretation is tentatively supported by the absence of a significant association between AV performance and Stroop inhibition control scores, a proxy for executive control.

Moberly and colleagues (15) demonstrated that preoperative AV sentence recognition performance predicted auditory-only sentence recognition outcomes 6 months after cochlear implantation. This finding suggested that the ability to effectively process visual speech cues may support the restoration of auditory language processing. Thus, visual speech input may promote or preserve functional language networks during auditory deprivation. To investigate the cognitive-linguistic factors underlying ongoing effective AV processing before cochlear implantation, this study identified STM as a strong predictor of AV sentence recognition in CI candidates. These results suggest that STM may help explain why some individuals benefit more from visual input in multisensory speech tasks and point to its potential role in supporting auditory language outcomes following implantation.

More broadly, findings of this study have several clinically translational areas of relevance. First, as noted above, prior work has demonstrated that AV sentence recognition may serve to help predict postoperative CI auditory-only speech recognition performance (15). Thus, assessing AV sentence recognition in the preoperative setting may help clinicians to provide better counseling regarding expected CI outcomes (likely in combination with other patient factors). Understanding the factors that contribute to preoperative AV sentence recognition abilities, including STM abilities, may help provide a more complete picture of the underlying abilities of CI candidates and their contributions to predicting CI outcomes. Additionally, there is some evidence that memory can be improved through focused cognitive training approaches, especially those that include cognitive training in conjunction with auditory training (29,30). Although the topic of the efficacy of memory training is well beyond the scope of the current study, it is reasonable to suggest that the role of STM in auditory and AV speech recognition outcomes deserves additional research.

This study has several limitations. First, we did not directly assess participants’ visual-only speechreading ability. Although prior studies in CI candidates have typically shown near-floor performance on visual-only speech recognition, future studies should incorporate a dedicated visual-only condition to isolate the contributions of visual, auditory, and combined AV processing (15). Second, 2 participants did not complete the full battery of cognitive-linguistic assessments, likely due to fatigue near the end of testing. Missing data may have affected the reported correlations. Another limitation includes the relatively small sample size, which precluded the use of multivariable regression models to examine the independent and combined contributions of cognitive-linguistic variables. We were also unable to control for participants’ listening configurations (eg, unilateral vs bilateral hearing aid use vs no hearing aids), which may influence AV processing strategies, but we did account for performance differences among listeners with differing listening configurations by controlling for their AzBioQ sentence recognition performance in their everyday listening configurations in our analyses. Future studies would benefit from testing all participants in the best-aided bilateral hearing configuration. Furthermore, although age was not significantly correlated with MLST scores, our limited statistical power prevented a more thorough examination of age-related effects.

## CONCLUSION

This study demonstrates that AV sentence recognition in adults with moderate-to-profound SNHL is not explained solely by traditional demographic or audiologic variables. Instead, performance was predicted by STM capacity, as measured by Forward Digit Span, while accounting for auditory-only sentence recognition abilities. Other cognitive-linguistic measures were not significantly related to AV sentence recognition performance. Unlike conventional auditory-only tests, the MLST more closely approximates real-world communication demands by engaging both auditory and visual systems. In doing so, it helps reveal cognitive predictors of AV speech recognition abilities, which are relevant to much of everyday communication.

## FUNDING SOURCES

This study was supported by the National Institutes of Health, National Institute on Deafness and Other Communication Disorders (NIDCD) R01DC019088 to A.C.M. and R21DC019382 and the National Institute on Aging R01AG089200 to T.N.T.

## CONFLICTS OF INTEREST

Author A.C.M. serves as CMO and on the Board of Directors for Otologic Technologies and as a paid consultant for Amgen. Author T.N.T. has received grant funding from Cochlear Americas. For other authors, none declared.

## DATA AVAILABILITY STATEMENT

Data used in this study are available upon request from the corresponding author.

## References

[1] Holden LK Finley CC Firszt JB. Factors affecting open-set word recognition in adults with cochlear implants. Ear Hear. 2013;34:342–360. 23348845 10.1097/AUD.0b013e3182741aa7 PMC3636188

[2] James CJ Karoui C Laborde ML. Early sentence recognition in adult cochlear implant users. Ear Hear. 2019;40:905–917. 30335668 10.1097/AUD.0000000000000670

[3] Lenarz M Sönmez H Joseph G Büchner A Lenarz T. Long-term performance of cochlear implants in postlingually deafened adults. Otolaryngol Head Neck Surg. 2012;147:112–118. 22344289 10.1177/0194599812438041

[4] Boisvert I Reis M Au A Cowan R Dowell RC. Cochlear implantation outcomes in adults: a scoping review. PLoS One. 2020;15:e0232421. 32369519 10.1371/journal.pone.0232421 PMC7199932

[5] Bierbaum M McMahon CM Hughes S. Barriers and facilitators to cochlear implant uptake in Australia and the United Kingdom. Ear Hear. 2020;41:374–385. 31356385 10.1097/AUD.0000000000000762

[6] McRackan TR Hand BN Velozo CA Dubno JR; Cochlear Implant Quality of Life Consortium. Validity and reliability of the Cochlear Implant Quality of Life (CIQOL)-35 profile and CIQOL-10 Global instruments in comparison to legacy instruments. Ear Hear. 2021;42:896–908. 33735907 10.1097/AUD.0000000000001022 PMC8222065

[7] Moberly AC Vasil KJ Ray C. Visual reliance during speech recognition in cochlear implant users and candidates. J Am Acad Audiol. 2020;31:30–39. 31210633 10.3766/jaaa.18049 PMC6911035

[8] Stevenson RA Sheffield SW Butera IM Gifford RH Wallace MT. Auditory-visual speech recognition by hearing-impaired subjects: consonant recognition, sentence recognition, and auditory-visual integration. Ear Hear. 2017;38:521–538. 28399064 10.1097/AUD.0000000000000435 PMC5570631

[9] Grant KW Walden BE Seitz PF. Auditory-visual speech recognition by hearing-impaired subjects: consonant recognition, sentence recognition, and auditory-visual integration. J Acoust Soc Am. 1998;103:2677–2690. 9604361 10.1121/1.422788

[10] Kushalnagar RS Vogler C. Teleconference accessibility and guidelines for deaf and hard of hearing users. In: Proceedings of the 22nd International ACM SIGACCESS Conference on Computers and Accessibility. ACM; 2020:1–6.

[11] Rouger J Lagleyre S Fraysse B Deneve S Deguine O Barone P. Evidence that cochlear-implanted deaf patients are better multisensory integrators. Proc Natl Acad Sci U S A. 2007;104:7295–7300. 17404220 10.1073/pnas.0609419104 PMC1855404

[12] Gao Y Idemaru K Werker JF Joanisse MF. Audiovisual integration in spoken language processing: a neurocognitive perspective. Neurosci Biobehav Rev. 2021;127:709–720.34058557

[13] Moberly AC Du L Tamati TN. Individual differences in the recognition of spectrally degraded speech: associations with neurocognitive functions in adult cochlear implant users and with noise-vocoded simulations. Trends Hear. 2025;29:1–21. 10.1177/23312165241312449 PMC11742172 39819389

[14] Frei V Schmitt R Giroud N. Processing of visual speech cues in speech-in-noise comprehension depends on working memory capacity and enhances neural speech tracking in older adults with hearing impairment. Trends Hear. 2024;28:23312165241287622. 10.1177/23312165241287622 PMC11520018 39444375

[15] Moberly AC Tamati TN Pisoni DB. Audiovisual processing skills before cochlear implantation predict postoperative speech recognition in adults. Ear Hear. 2023;45:617–625. 38143302 10.1097/AUD.0000000000001450 PMC11025067

[16] Kirk K Prusick L French B. Evaluating multimodal speech perception in adults with cochlear implants and hearing aids. Paper presented at: 12th Conference on Cochlear Implant and Other Implantable Auditory Technology. Baltimore; 2012.

[17] Lewis JH Castellanos I Moberly AC. The impact of neurocognitive skills on recognition of spectrally degraded sentences. J Am Acad Audiol. 2021;32:528–536. 34965599 10.1055/s-0041-1732438 PMC11740587

[18] Spahr AJ Dorman MF Litvak LM. Development and validation of the AzBio sentence lists. Ear Hear. 2012;33:112–117. 21829134 10.1097/AUD.0b013e31822c2549 PMC4643855

[19] Wilkinson GS Robertson GJ. Wide Range Achievement Test. 4th ed. Lutz FL: Psychological Assessment Resources; 2006.

[20] Nittrouer S Burton LT. The role of early language experience in the development of speech perception and phonological processing abilities: evidence from 5-year-olds with histories of otitis media with effusion and low socioeconomic status. J Commun Disord. 2005;38:29–63. 15475013 10.1016/j.jcomdis.2004.03.006

[21] Raven JR Court JH. Manual for Raven’s Progressive Matrices and Vocabulary Scales. Oxford: Oxford Psychologists Press; 1998.

[22] Wechsler D. WISC-IV: Wechsler Intelligence Scale for Children, Integrated: Technical and Interpretive Manual. Hancourt Brace and Company; 2004.

[23] Stroop JR. Studies of interference in serial verbal reactions. J Exp Psychol Anim Learn Cogn. 1935;18:643–662.

[24] Torgesen JK Rashotte CA Wagner RK. TOWRE: Test of Word Reading Efficiency. Pro-ed; 1999.

[25] Cohen J. Statistical Power Analysis for the Behavioral Sciences. Routledge; 2013.

[26] Gao C Green JJ Yang X Oh S Kim J Shinkareva SV. Audiovisual integration in the human brain: a coordinate-based meta-analysis. Cereb Cortex. 2023;33:5574–5584. 36336347 10.1093/cercor/bhac443 PMC10152097

[27] Walden BE Grant KW Cord MT. Effects of amplification and speechreading on consonant recognition by persons with impaired hearing. Ear Hear. 2001;22:333–341. 11527039 10.1097/00003446-200108000-00007

[28] Rönnberg J Lunner T Zekveld A. The Ease of Language Understanding (ELU) model: theoretical, empirical, and clinical advances. Front Syst Neurosci. 2013;7:31. 23874273 10.3389/fnsys.2013.00031 PMC3710434

[29] Castiglione A Benatti A Velardita C. Aging, cognitive decline and hearing loss: effects of auditory rehabilitation and training with hearing aids and cochlear implants on cognitive function and depression among older adults. Audiol Neurootol. 2016;21:21–28. 27806352 10.1159/000448350

[30] Ferguson MA Henshaw H. Auditory training can improve working memory, attention, and communication in adverse conditions for adults with hearing loss. Front Psychol. 2015;6:556. 26074826 10.3389/fpsyg.2015.00556 PMC4447061

